# Examining equity in access to long-lasting insecticide nets and artemisinin-based combination therapy in Anambra state, Nigeria

**DOI:** 10.1186/1471-2458-12-315

**Published:** 2012-05-22

**Authors:** Chinyere O Mbachu, Obinna E Onwujekwe, Benjamin SC Uzochukwu, Eloka Uchegbu, Joseph Oranuba, Amobi L Ilika

**Affiliations:** 1 Health Policy Research Group, Department of Pharmacology and Therapeutics, College of Medicine, University of Nigeria, Enugu, Enugu State, Nigeria; 2 Department of Community Medicine, University of Nigeria Teaching Hospital, Enugu, Enugu State, Nigeria; 3 Department of Health Administration and Management, Faculty of Health Sciences, University of Nigeria, Enugu, Enugu State, Nigeria; 4 Ministry of Health, Awka, Anambra State, Nigeria

**Keywords:** Malaria, Equity, Long-lasting insecticide nets, LLIN, ACT, Artemisinin-based combination therapy

## Abstract

**Background:**

In order to achieve universal health coverage, the government of Anambra State, southeast Nigeria has distributed free Long-lasting Insecticide treated Nets (LLINs) to the general population and delivered free Artemisinin-based Combination Therapy (ACT) to pregnant women and children less than 5 years. However, the levels of coverage with LLINS and ACTs is not clear, especially coverage of different socio-economic status (SES) population groups. This study was carried out to determine the level of coverage and access to LLINs and ACTs amongst different SES groups.

**Methods:**

A questionnaire was used to collect data from randomly selected households in 19 local government areas of the State. Selected households had a pregnant woman and/or a child less than 5 years. The lot quality assurance sampling (LQAS) methodology was used in sampling. The questionnaire explored the availability and utilization of LLINs and ACTs from 2394 households. An asset-based SES index was used to examine the level of access of LLINS and ACTs to different SES quintiles.

**Results:**

It was found that 80.5 % of the households had an LLIN and 64.4 % of the households stated that they actually used the nets the previous night. The findings showed that 42.3 % of pregnant women who had fever within the past month received ACTs, while 37.5 % of children ≪5 years old who had malaria in the past month had received ACTs. There was equity in ownership of nets for the range 1–5 nets per household. No significant SES difference was found in use of ACTs for treatment of malaria in children under five years old and in pregnant women.

**Conclusions:**

The free distribution of LLINs and ACTs increased household coverage of both malaria control interventions and bridged the equity gap in access to them among the most vulnerable groups.

## Background

Malaria is the most significant public health problem in Nigeria [1,2]. The disease is responsible for 60 % outpatient visits to health facilities, 30 % childhood death, 25 % of death in children under one year, 11 % maternal death [3]. The financial loss due to malaria annually is estimated to be about 132 billion Naira in form of treatment costs, prevention, loss of man-hours etc, yet, it is a preventable and treatable disease [3].

The sixth Millennium Development Goal (MDG 6), which is to combat HIV/AIDS, Malaria and other diseases, has among its second target to have halted by 2015 and begun to reverse the incidence of malaria. Two of the indicators for this goal are the proportion of children under 5 sleeping under insecticide-treated bed nets and the proportion of children under 5 with fever who are treated with appropriate anti-malarial drugs [4].

Malaria control in Nigeria anchors on the multi-pronged global strategies for malaria control. These include prompt and effective case management (early diagnosis and prompt treatment with effective drugs - artemisinin-based combination therapy); intermittent preventive treatment of malaria in pregnancy (IPTp); Integrated Vector Management including use of Long-lasting Insecticide Treated Nets (LLINs); Indoor Residual Spraying (IRS) and Environmental Management [3].

ITNs are a key prevention tool that have been found to reduce uncomplicated malaria in children by 51 % and decrease all-cause mortality by 18 % in children aged 1–59 months [5]. By preventing malaria, LLINs reduce the need for treatment and the pressure on health services [6]. The MDG report of 2011 indicated a remarkable surge in the production, purchase and distribution of insecticide-treated mosquito net globally, and particularly in Africa, with a marked increase in ownership and use among children, that were attributed to widespread nationwide campaigns for the distribution of free nets [7].

The Nigerian National Demographic and Health Survey (NDHS) of 2008 recorded 8 % household coverage with insecticide-treated nets (ITNs) and this was seen to increase with wealth quintiles [8]. Some studies have highlighted poverty as a barrier to scaling up of ITN in Nigeria since a large proportion of the population still live under a dollar per day [9,10]. Even when mass distribution of free nets is attempted, the limited resources of the country considering the very large population, makes sustainability almost impossible without continuous support from donor agencies [10].

Following the extensive resistance to chloroquine and sulphadoxine-pyrimethamine in Nigeria, ACTs were introduced in 2005 as the first line anti-malaria drug of choice [11]. Three years after this introduction, the NDHS showed that an average of 2.5 % of children under five years who had fever were treated with ACTs and this increased significantly with wealth quintile and mother’s education [8]. Cost and frequent stock out of ACTs in public health facilities have been recorded as the major reasons for low coverage rates [12]. The National policy on diagnosis and treatment of malaria states that the recommended medicines for treatment of uncomplicated malaria in all age groups are ACTs, except for children ≪5 kg and pregnant women in first trimester, and that all medicines provided by The Federal Government to public health facilities shall be at no cost to the clients [13]. Whilst limiting as far as possible the inappropriate use by those who do not have malaria, there is a need to ensure that these commodities reach those who are most at-risk [14].

In order to improve access to LLINs and ACTs, the government of Anambra State, southeast Nigeria under the National Malaria Control Booster Programme embarked on free mass distribution LLINs and provision of free ACTs in public primary health facilities, with the aim of ensuring wide-scale and equitable deployment of LLINs and ACTs amongst other malaria control tools in the state. The LLINs were distributed through routine distribution methods and campaigns and ACTs were supplied to primary and secondary public health facilities. The State Malaria Control Booster programme started distributing LLINs and ACTs to pregnant women and children <5 years since January 2010, but the level of coverage of the programme, especially as it relates to different socio-economic status (SES) groups is not clear. Though infrequently conducted, household surveys are the preferred means of assessing whether or not sufficient LLINs and ACTs have been delivered and utilized to cover population at risk of malaria, and to provide up to date estimates [15].

The paper provides new information about the level of coverage that a free distribution of LLINs and ACTs was able to achieve. The findings will be useful for decision-makers to understand the level of coverage that free malaria control tools can achieve and to develop strategies that will ensure proper coverage of these interventions.

## Methods

### Study area and design

The study was conducted in Anambra state, south-east Nigeria in April 2011. Anambra state has a total of 21 local government areas (LGAs) and a population of 4,182,032 inhabitants whose major occupations are farming (75 %), trading and fishing [16]. Malaria transmission is stable all year round but higher rates are experienced during the rainy months. Pregnant women and children are the most affected by malaria in the state. There are about 382 primary health centers (PHCs), owned and managed by Local Government Areas (LGAs) in the State.

**Figure 1:**
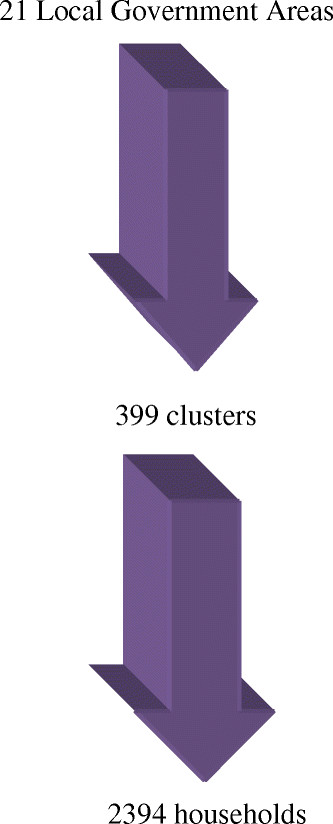
Flowchart showing sample selection process from local government areas to households.

Lot quality assurance sampling (LQAS) method was used [17]. 19 clusters were selected from each of the 21 LGAs. For the purpose of this study, a cluster was defined as a group of households served by a public health facility that provides primary level of care. In each of the clusters, households were listed, a total of six households that met the inclusion criteria of having a pregnant woman and/or a child less than five years living in the house were picked by simple random sampling, and only one respondent was interviewed in each household. A total of 2394 households were selected but the sample size was increased to 2500 (Figure 1). Questionnaires were administered to a random selection of respondents who were in order of preference, pregnant women, mothers of children less than five years old, female caregivers, male caregivers. In the absence of an informant, the household was replaced by simple random sampling with another household.

Data was collected from the respondents by trained interviewers using a questionnaire. Information was collected on personal data/demographics of respondents and their household members; availability and use of LLINs; source of LLINs; use of ACTs with emphasis on pregnant women and children less than five years old; health facility visited for treatment of malaria; and cost of treatment of malaria in pregnant women and children ≪5 years. Information was also collected on the household food expenditure and assets.

### Data analysis

Data was analyzed using the statistical package for social sciences version 15 (SPSS 15). The questionnaires were coded for identification and each one was entered into the system with its code. Data entry was done once by the data clerks and double checked by the researchers. Frequencies were calculated for all the variables to determine missing values and further clean the data.

Frequencies were calculated for household ownership and utilization of LLINs to determine its coverage. Frequencies were also calculated for utilization of ACTs for treatment of fever in pregnant women and children less than five years old. The socio-economic status (SES) of the households was determined using an-asset based index, which was computed using principal components analysis. The SES index was used to divide the sample into quintiles. Ownership and utilization of LLINs, as well as the utilization of ACTs were disaggregated into SES quintiles. Where appropriate, chi square test was used for tests of significance for proportions of categorical variable, and all test of significance were done based on a p-level of 0.05.

### Ethical approval

Ethical approval was obtained from the Health Research Ethics Committee of University of Nigeria Teaching Hospital, Enugu State, Nigeria.

## Results

Data was collected from 2401 respondents giving a response rate of 96 %.

Table 1 shows the results of demographic characteristics of the respondents. 2112 (88 %) were females, 1348 (56.2 %) were within age group 25–34 years with mean age of 31.8 years. The results also showed that 898 (37.1 %) of our respondents were pregnant women with mean gestational age of 5.6 months (SD 2.0). 2234 (95.8 %) of households surveyed had at least one child under five years old, and 1578 (65.6 %) of the surveyed households had between 5 and 10 members living in the house (Table 1).

**Table 1:** Socio-demographic characteristics of respondents

**Characteristics**	**Proportions n (%)**
Sex	
·Females	2112 (88)
·Males	287 (12)
Age (years)	
·15–24	313(13.1)
·25–34	1347(56.2)
·35–44	600(25.1)
·45–54	106(4.4)
·55–64	24(1.0)
·65–74	5(0.2)
Mean Age (years) = 31.8(SD 7.3)	
Number of pregnant women	898(37.1)
Mean Gestational Age (months) = 2.1(SD 2.9)	
Number of households with at least one child ≪5 years	2300 (95.8)
Number of households with no child ≪5 years old	101(4.2)
No. of People in Households (N = 2397)	
·≪5	808(33.6)
·5–10	1574(65.6)
·>10	19(0.8)

Table 2 shows that 1932 (80.5 %) of households had LLINs and the mean number of nets per household was two. On source of LLINs the study showed that 2187 (81.1 %) of households who had LLINs received the nets during the free distribution while 77 (3.2 %) purchased theirs. A small proportion of the respondents had got their LLINs from relatives, friends or during election campaigns. Of the 469 (19.5 %) of households that did not have LLINs, reasons for not having included: didn’t receive during free distribution- 369 (78.7 %); don’t know where to get LLINs- 66 (14.1 %); don’t have money for LLINs- 64 (13.6 %); don’t think it is important 49 (10.4 %); don’t like it- 32 (6.8 %). Other reasons which made up the rest (46/469 i.e. 9.8 %) were: new to place or just moved to the place; left net in former place of residence; was not pregnant during period of distribution; was not around during distribution; there was a lot of rush during distribution; nets got damaged; shared to women alone; not necessary- mosquitoes don’t bite me, I have a plant that kills insects, mosquitoes are not in this village.

**Table 2:** Ownership and utilization of LLINs by members of households and reasons for non-utilization

Variables	Proportions **n(%)**
Number of households that have a LLIN	1932 (80.5)
**Mean number of LLINs per household = 2**	
Number of households where a LLIN was used the previous night	1244 (64.4)
Number of households in which a LLIN was used the previous night by: (N_1_ = 1244)	
·All household members	784 (63.0)
·Pregnant women only	83 (6.7)
·Children under five years only	377 (30.3)
Reasons for non-use of LLINs by households (N_2_ = 688)	
·Forgot to use it	21 (3.1)
·Makes me sweat	454 (66.0)
·Not necessary	43 (6.3)
·Mosquitoes still bite me	51 (7.5)
·Skin irritation	65 (9.4)
·Other reasons	65 (9.4)

N_1_ represents number of households where a long-lasting insecticide treated net was used the previous night which is 1244 households.

N_2_ represents number of households where a long-lasting insecticide treated net was not used the previous night, which is 688 households.

For utilization of the LLINs, Table 2 shows that 1546 (64.4 %) of the households had members who had slept under their LLINs the previous night, and in 964 (65 %) of these households, all members slept under the nets. Of those who had not used nets the previous night, based on a multiple response question on reasons for non use, 454 (66 %) said it made them sweat, while 43 (6.3 %) said LLIN is not a necessary preventive measure for malaria (Table 2).

The result also shows that 965 (40.2 %) of surveyed households had at least one case of fever in the past one month, with the highest distribution of fever, 446 (46.2 %), being in children under five years. The proportions of children under five and pregnant women with fever are 446/2234 (20 %) and 227/898 (25.3 %) respectively. Only 96 (42.3 %) of the pregnant women with fever and 167 (37.5 %) children under-five years with fever sought care at a primary health care facility. Of those treated, 45.7 % of pregnant women and 38.7 % of children under-five years were treated with ACTs (Table 3).

**Table 3:** Drugs used in primary health care facilities for treatment of malaria in pregnant women and children less than five years

Drugs used for treatment of malaria	Proportion of people treated **n (%)**
**1.**Pregnant women (N = 380)	
Artemisinin-based combination therapy (ACT)	174 (45.7)
Chloroquine	1 (0.4)
Quinine	8 (2.2)
Sulphadoxine-Pyrimethamine (SP)	129 (33.9)
Artesunate monotherapy (AM)	4 (1.1)
Others	64 (16.7)
**2.**Children less than five years (N = 838)	
Artemisinin-based combination therapy	324 (38.7)
Chloroquine	127 (15.2)
Quinine	27 (3.2)
Sulphadoxine-Pyrimethamine	86 (10.2)
Artesunate monotherapy	28 (3.3)
Others	246 (29.4)

Others include herbal medicines and concoctions; unknown mixture of tablets and injections; paracetamol; and paludrine.

Table 4 shows that there is equity in ownership of LLINs for the range 1–5 nets per household, but the ownership of more than 5 LLINs is higher among the rich. Also, there was significant difference across SES quintiles among those who received nets during immunization, free distribution and those who purchased (Table 4).

**Table 4:** Socioeconomic differences in the ownership and source of LLINs

Variables	Q1 n(%)	Q2 n(%)	Q3 n(%)	Q4 n(%)	Q5 n(%)	Q1:Q5 (equity ratio)	Chi square (*p* value)
**Availability of LLINs in household**	365(78)	388(83)	393(84)	380(81)	359(77)	1.02	12.38(0.15)
**No. of LLINs per household**							
1–5	362(99.2)	385(99)	389(99)	369(97)	344(96)	1.05	
>5	3(0.8)	3(1)	4(1)	11(3)	15(4)	0.15	157.29(0.0)
**Source of LLIN**							
During immunization	38 (11)	56 (14)	70 (18)	60 (18)	79(21)	0.52	15.92(0.003)
Antenatal clinics	79 (22)	78 (20)	88 (22)	60 (18)	67(18)	1.22	7.78(0.100)
Free distribution	288 (78)	295 (75)	313 (80)	330 (87)	304(89)	0.98	17.21(0.002)
Purchased LLIN	13(4)	3 (1)	15 (4)	10 (3)	19(5)	0.80	12.78(0.012)
Others	6 (1.7)	1 (0.3)	4 (1)	4 (1)	1(0.3)	5.67	6.53(0.160)

Table 5 shows the SES differences in the utilization of antimalarials for prevention and treatment of malaria by pregnant women and children less than five years across wealth quintiles. There was no significant difference in the utilization of antimalarials for treatment of malaria in children ≪5 years and in pregnant women but for ‘others’ in prevention of malaria among pregnant women.

**Table 5:** Socioeconomic differences in the utilization of antimalarials by pregnant women

Antimalarials used for treatment of fever	Q1 (%)	Q2 (%)	Q3 (%)	Q4 (%)	Q5 (%)	Q1:Q5	Chi square (*p* value)
**Children less than five years**							
ACTs	9(28)	13(29)	13(33)	29(43)	27(26)	1.1	5.97(0.20)
Chloroquine	0(0)	1(2)	0(0)	0(0)	2(2)	0	2.85(0.58)
Quinine	0(0)	0(0)	1(2.6)	1(1.5)	1(1)	0	1.83(0.77)
Sulfadoxine-pyrimethamine	7(21)	11(24)	8(21)	13(19)	22(21)	1	0.42(0.98)
Artesunate monotherapy	1(3)	2(4)	5(13)	4(6)	9(9)	0.3	3.69(0.45)
Others	5(15)	4(9)	7(18)	7(10)	6(6)	2.5	5.61(0.23)
**Pregnant women for treatment of malaria**							
ACTs	10(27)	16(28)	15(34)	36(48)	42(38)	0.71	7.66(0.11)
Chloroquine	0(0)	0(0)	0(0)	0(0)	1(1)	0.0	1.94(0.75)
Quinine	1(3)	0(0)	1(2.3)	1(1.3)	2(2)	1.49	1.45(0.84)
Sulfadoxine-pyrimethamine	10(27)	21(37)	14(32)	16(21)	30(27)	0.99	4.21(0.38)
Artesunate monotherapy	0(0)	1(2)	0(0)	1(1)	1(1)	0.0	1.32(0.86)
Others	10(27)	5(9)	11(25)	12(16)	6(5)	4.95	18.09(0.001)

## Discussion

Although the finding of 80.5 % LLINs coverage rate in the households sampled meets the RBM target and is a significant improvement on the NDHS of 2008, it falls short of the country’s roadmap implementation target for 2010, which is a 95 % coverage rate [8,18]. According to the 2008 NDHS report, 8 % of surveyed households owned at least one insecticide treated net (ITN), 3 % owned >1 ITN, and the average number of ITNs per household is ≪1. Ownership of ITNs was higher among households in the Southern zones and increased with wealth quintiles [8]. In the NMCP, free LLINs are given only to pregnant women and children ≪5 years and the target of RBM is for 80 % coverage of LLINs by 2010. This improvement, however, can be attributed to the State-wide free LLINs distribution that took place about a year ago, since 81.1 % of those who owned LLINs had obtained them during the free distribution exercise. The shortfall could be accounted for by the abrupt halt in the free distribution exercise since majority of the respondents who did not have the nets were absent at the time of distribution. Lack of funding and the size of at risk population to be reached have been identified as major restricting factors to complete coverage of LLINs [12].

Household ownership of LLINs was found to be equitably distributed across all quintiles as opposed to the 2008 NDHS which found that households in higher wealth quintiles significantly owned ITNs more than those in lower quintiles [8]. The implication of this finding is that free LLINs distribution minimized inequalities in ownership of LLINs by enabling households in the poorest wealth quintiles to access this malaria control strategy. Other studies in African countries have revealed similar findings of decreased inequity following distribution of free ITNs [9,19-22]. However, we find that there is significant equity difference in the ownership of more than 5 LLINs per household. Controversy still exists in this aspect as some studies have found that lower wealth quintiles owned more number of nets per household than higher ones, depending on the setting of the study, rural or urban [11,19].

Over half of the households in this survey had members who slept under the LLINs the previous night. This is an improvement on the 2008 NDHS which found 49.8 % and 44.4 % utilization rates among children ≪5 years and pregnant women respectively, but it still falls short of the RBM target for 2010 which is an 80 % utilization rate [1,8]. Among our respondents, the commonest reason for non-use of LLINs was that it made them sweat( 66 %), as opposed to a study in 2008, where 71.4 % of non-use was as a result of lack of awareness about LLINs and only 3.4 % for reason of sweating [23]. This difference in reason for non-use could be explained by the increased utilization rates, since only those who use nets can experience sweating under them. Although a positive association can be drawn between LLIN ownership and use, we cannot from our study conclusively say that there is a causal relationship. Several studies in Africa have also found a positive relationship between LLINs ownership and use among households [19-22].

Although ACTs were the most utilized for treatment of malaria, the utilization rates falls far short of the RBM target of 80 % by 2010 [24]. There seems to be decrease in utilization of ACTs from our study when compared to a cross-sectional multi country survey on coverage rate of ACTs which found 52 % coverage rate among children in Nigeria in 2007 [25]. This shortfall could be explained by the inconsistencies in the delivery of ACTs to the public health facilities which is being experienced in Anambra State. A quality assessment survey done by the Health Policy Research Group in November 2010 showed that 54.4 % of the primary health care facilities in Anambra State had been out of stock for ACTs for a period of at least 3 months [26]. Studies in Africa have found that a large proportion of patients seek care at public health facilities first, and when they cannot access this care they resort to sub standard care for the reason of cost [27]. This was consistent with our finding of the continued use of less efficacious antimalarials like chloroquine and Sulphadoxine-pyrimethamine which have high levels of drug resistance in Africa. Of greater concern is the use of artemisinin monotherapy (AM). Considering that the cost of one adult dose of the first choice ACT (Artemisinin-Lumefantrine) was calculated to be US$10.3 [28], which is high especially where the mean total food expenditure as found in our survey is 6,785.23 (US$45.2), and the potential threats of drug resistance which AM poses to the only currently proven efficacious antimalarial (ACTs), there is need to scale-up free ACTs.

Despite the low utilization rate of ACTs, there was equity in access among the most vulnerable group, children less than five years old and pregnant women. Inconsistencies in delivery of free ACTs to public health facilities and cost of purchasing them when free ones are not available have been identified as the two major barriers to scaling up ACTs coverage rate among vulnerable groups. Subsidizing ACTs by 90 % was found to increase the proportion of consumers purchasing them for a significantly higher number of under-fives from 1 % to 44 % in one year in Tanzania [29]. The fact that ACTs were provided free of charge in the public health facilities could explain the bridge in equity gap in access between the rich and the poor.

Free distribution of LLINs significantly increased its coverage rate and reduced inequities in ownership of nets across wealth quintiles. A positive association can be drawn between nets ownership and use. Distribution of free LLINs to all households is likely to achieve the goal of combating malaria regardless of socioeconomic status.

The free distribution exercise did not significantly improve ACT coverage among vulnerable groups, although inequities do not exist in the access to ACTs by the most vulnerable group, children less than five years. Sustainability and cost have been identified as major limiting factors to ACTs coverage.

Success of malaria control requires strong, sustained political and budgetary commitment. Policy makers and parliamentarians need to support the malaria control programs and health systems in their countries by providing and coordinating the necessary resources, especially financial; by helping to resolve bottlenecks in countries (e.g. taxes and tariffs, administrative or regulatory procedures); and coordinating all partners active in the country to reach rapidly the universal coverage targets.

Sustainability is the key to achieving lasting results. Behavior change communication approach will be an important tool to address the 10 % of respondents who still think use of LLINs is not an important malaria prevention strategy.

The main limitation of the study is that it is cross-sectional, not a randomized controlled trial. Therefore, strong cause and effect relationships cannot be established. The study was conducted less than one year after the roll out of free LLINs, so the increased coverage and use may be transient, and there is likelihood that it may drop with time as ITNs wear out. There may be a need for further studies with longer follow up periods to ascertain the sustainability of current coverage rates.

However, the strength of this study is that respondents (households) were selected randomly and therefore make a good representation of the study population. This implies that the results of the study will be reproduced if the whole population is used.

This study provides some information on how the national and international target of 80 % coverage and use of LLINs might be achieved, as well as reducing inequality through free distribution. Further research with an ideal randomized controlled design with sufficient households and time for follow up, should clarify the possible long-term impact of changing distribution policies from cost-sharing schemes to free distribution.

## Conclusion

The free distribution of LLINs and ACTs increased coverage of both malaria control interventions compared to previous surveys (NDHS, 2008). Coverage rate of LLINs slightly exceeded the Roll Back Malaria target, but this was not so for coverage of ACTs. It also bridged the equity gap in access to these malaria control interventions. Although there was increase in ACTs coverage compared to figures from 2008 National Demographic and Health Survey (NDHS) which found a 2.4 % coverage rate for the country, there needs to be a sustained effort to ensure that the rate of increase is maintained till the RBM target is achieved.

## Competing interests

The authors declare that they have no competing interests.

## Authors’ contributions

OEO, BSCU, JO and AI conceived the study. All the authors participated in data collection and analysis. CM, OEO and EU drafted the manuscript. All the authors participated in revising the manuscript and agreed to the contents of the final version. All authors read and approved the final manuscript.

## Funding

The study was funded by the Anambra State Ministry of Health.

## Pre-publication history

The pre-publication history for this paper can be accessed here:

http://www.biomedcentral.com/1471-2458/12/315/prepub
